# Long-Term Consequences of Fetal Angiotensin II Receptor Antagonist Exposure

**DOI:** 10.1155/2018/5412138

**Published:** 2018-01-11

**Authors:** K. Wegleiter, M. Waltner-Romen, R. Trawoeger, U. Kiechl-Kohlendorfer, E. Griesmaier

**Affiliations:** Department of Pediatrics II, Neonatology and Neonatal Intensive Care Unit, Medical University of Innsbruck, Innsbruck, Austria

## Abstract

Fetal angiotensin II receptor antagonist exposure is associated with major complications and even death when administered during pregnancy. Neonates frequently require intensive care treatment, and mortality is high. Despite this well-known risk potential, a considerable number of women still receive angiotensin II receptor antagonists during pregnancy to treat arterial hypertension. Although clinical symptoms in the neonatal period are well described, few reports address long-term follow-up after fetal exposure to angiotensin II receptor antagonists. We here report on a patient who was unwittingly exposed to olmesartan medoxomil during pregnancy. After birth, the neonate presented with mild clinical symptoms, mainly affecting the kidneys. However, neurodevelopmental follow-up revealed a delay in motor development with muscular hypotonia and failure to thrive at age 2 years. This case highlights the fact that, despite not causing neurological symptoms in the neonatal period, fetal angiotensin II receptor antagonist exposure during pregnancy might lead to neurodevelopmental impairment in later life.

## 1. Introduction

Maternal hypertension is a frequent condition and is associated with major fetal and maternal complications when not treated [1]. Methyldopa, beta blockers, and calcium channel blockers are the drugs of choice during pregnancy. In nonpregnant women, angiotensin-converting enzyme inhibitors and angiotensin II receptor antagonists are popular first-line therapies for primary hypertension. However, administration of angiotensin II receptor antagonists is strongly contraindicated in the second and third trimesters and is also controversially discussed in the first trimester of pregnancy [2]. Despite the well-known risk potential for drug-related fetopathy, a considerable number of women still receive angiotensin II receptor antagonists during pregnancy, thus making it important to report on this issue in order to avoid additional case pathologies. Neonates who were exposed to angiotensin II receptor antagonists during the fetal period develop renal impairment due to abnormal kidney development. Fetuses suffer from oligohydramnios, leading to limb contractions and pulmonary hypoplasia, premature birth, growth retardation, and hypoplastic calvaria. Most of the affected infants require intensive care, including mechanical ventilation and peritoneal dialysis, because of lung hypoplasia and renal failure. Mortality rates are high, and consequently data on long-term outcome are scarce.

## 2. Case Presentation

**Figure 1:**
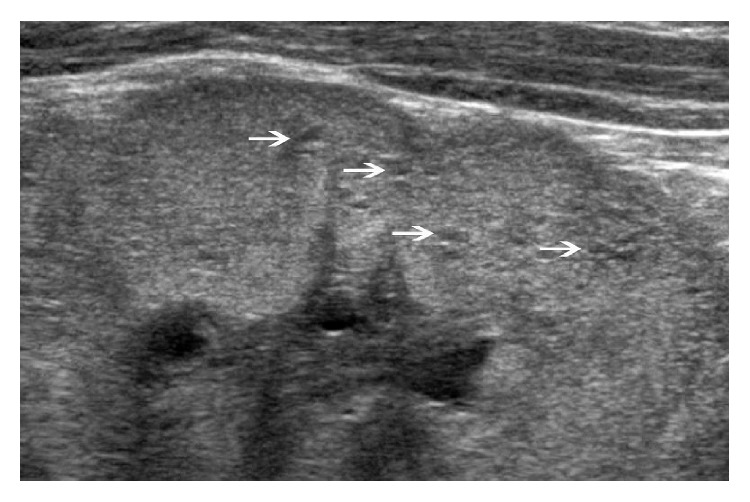
Ultrasound image of hyperechogenic kidney structure with multiple small cysts (white arrows) in the renal parenchyma.

We here report on a female patient with angiotensin II receptor antagonist-related fetopathy. Our patient is the first child of a 35-year-old mother. At a gestational age of 26 weeks, the mother was referred to our obstetrical center because of oligohydramnios and hyperechogenic fetal kidneys. Already before and during the pregnancy, she was treated with 5 mg olmesartan medoxomil, an angiotensin II receptor antagonist, because of grade 1 essential hypertension without evidence of end-organ damage. Maternal blood pressure showed values within the upper normal range, and placental blood flow and structure as well as serological screening for intrauterine infections were normal. Angiotensin II receptor antagonist-related fetopathy was suspected, and treatment was changed to a beta blocker (metoprolol) at 26 weeks 5 days' gestation. Subsequently, the amount of amniotic fluid increased to normal values, and further pregnancy was uneventful. A female term neonate was born at a gestational age of 39 weeks plus one day by caesarean section because of breech presentation. Birth weight was 2940 grams (16th percentile), head circumference 34 cm (25th percentile), and length 49 cm (20th percentile). Apgar scores were 7, 9, and 10 after 1, 5, and 10 minutes, respectively, and umbilical cord arterial pH was 7.25. A remarkable observation made during the first examination was a hypoplastic calvaria, expressed as huge fontanels and an asymmetry of the cranial bones. Cardiorespiratory function was normal, and blood pressure was within the normal range. Renal excretion was also normal, but the patient showed asymptomatic tubular proteinuria (protein quantitative/creatinine: 1687 mg/g) at the age of 4 days. Ultrasonography of the kidneys demonstrated a slight enlargement on both sides (length 4.9 cm, 74th percentile), a hyperechogenic structure with multiple small cysts, and decreased discrimination between renal cortex and parenchyma (Figure 1).

Family history regarding hereditary kidney diseases was unremarkable. Cranial ultrasound revealed normal findings, except for thalamostriatal vasculopathy, whereas analyses for neonatal infections including cytomegalovirus were negative. Magnetic resonance imaging of the brain and a detailed neurological examination were also normal. The patient was discharged home on day 9 of life. Proteinuria resolved within six months of age, while kidney cysts persisted. At follow-up, at age 9 months the patient presented with microcephaly (head circumference 44 cm, 4th percentile), muscular hypotonia, and motor delay. At age 26 months, she showed a failure to thrive and dystrophy (weight 9700 grams, below 3rd percentile; length 86 cm, 10–15th percentile). Hypotonia and delayed motor development were still present.

## 3. Discussion

We illustrate a case of angiotensin II receptor antagonist-related fetopathy with mild symptoms in the neonatal period. Our patient presented with typical features of angiotensin II receptor antagonist-related fetopathy such as oligohydramnios, fetal renal impairment, and hypocalvaria. From previous reports, this presentation with mild symptoms was rather unexpected, as it was previously shown that the critical time period is supposed to be around the 20th week of gestation [3]. Exposure of angiotensin II receptor antagonists during the second or third trimester of pregnancy is associated with deleterious outcome and high mortality rates [4]. Presumably due to the change in maternal treatment at the end of the second trimester, one might speculate that severe fetopathy could have been avoided in our case. Nevertheless, it is of interest that the patient developed neurodevelopmental impairment and dystrophy. This case underlines long-term neurodevelopmental delay in a newborn infant, suffering from angiotensin II receptor antagonist-related fetopathy with mild neonatal symptoms. Previously published reports focused mainly on neonatal transition and renal outcome, as the kidneys are the organs most frequently affected.

In agreement with previous studies, our postnatally performed neurological examinations showed normal results. In detail, neurological evaluation after birth, cerebral ultrasound, and magnetic resonance imaging were uneventful, especially not showing any signs of intrauterine hypoxia. However, it must be kept in mind that hypoplastic calvaria strongly indicates phases of arterial hypotonia and subsequent hypoxia during fetal development. In our patient, hypoplastic calvaria was severe; thus, one might assume that recurrent fetal hypoxia might still be the reason for developmental retardation later in life. Regarding failure to thrive, Miura and colleagues reported on two patients with severe growth retardation because of salt-losing diabetes insipidus. Both patients suffered from severe renal failure in the neonatal period, requiring peritoneal dialysis and mechanical ventilation. At age 2 and 6 years, polydipsia and salt-craving behaviour were observed [5]. In our case, there was no evidence for salt-losing diabetes insipidus, and the underlying cause for growth retardation is unclear. However, as sonography of the kidneys showed hyperechogenicity and multiple parenchyma cysts at age 2 years, normal renal outcome in future can be questioned.

The present case with motor impairment and failure to thrive at age 26 months underlines the importance of consistent interdisciplinary monitoring of all patients having been exposed to angiotensin II receptor antagonists during the fetal period, especially focusing on long-term neurodevelopmental outcome. Moreover, our case supports the standpoint that angiotensin II receptor antagonists should be strictly avoided during pregnancy.

## References

[1] Bateman B. T., Hernandez-Diaz S., Huybrechts K. F. (2012). Patterns of outpatient antihypertensive medication use during pregnancy in a Medicaid population. *Hypertension*.

[2] Moretti M. E., Caprara D., Drehuta I. (2012). The fetal safety of angiotensin converting enzyme inhibitors and angiotensin II receptor blockers. *Obstetrics and Gynecology International*.

[3] Oppermann M., Padberg S., Kayser A., Weber-Schoendorfer C., Schaefer C. (2013). Angiotensin-II receptor 1 antagonist fetopathy—risk assessment, critical time period and vena cava thrombosis as a possible new feature. *British Journal of Clinical Pharmacology*.

[4] Quan A. (2006). Fetopathy associated with exposure to angiotensin converting enzyme inhibitors and angiotensin receptor antagonists. *Early Human Development*.

[5] Miura K., Sekine T., Iida A., Takahashi K., Igarashi T. (2009). Salt-losing nephrogenic diabetes insipidus caused by fetal exposure to angiotensin receptor blocker. *Pediatric Nephrology*.

